# Air Quality and Environmental Injustice in India: Connecting Particulate Pollution to Social Disadvantages

**DOI:** 10.3390/ijerph18010304

**Published:** 2021-01-04

**Authors:** Jayajit Chakraborty, Pratyusha Basu

**Affiliations:** Department of Sociology & Anthropology, University of Texas at El Paso, El Paso, TX 79968, USA; pbasu@utep.edu

**Keywords:** air pollution, environmental justice, economic development, social inequalities, urbanization, India

## Abstract

While air pollution levels in India are amongst the highest in the world, the link between exposure to air pollution and social disadvantages has not been systematically examined. Using a distributive environmental justice framework, this study connects fine particulate matter (PM_2.5_) concentration data derived from satellite observations, a global chemical transport model, and ground-based measurements to district level socio-demographic information from the 2011 Census of India. The research objectives are to determine if annual average PM_2.5_ concentrations (2010) and recent increases in average PM_2.5_ concentrations (2010–2016) are unequally distributed with respect to socially disadvantaged population and household groups, after controlling for relevant contextual factors and spatial clustering. Overall, more than 85% of people and households in India reside in districts where international air quality standards for PM_2.5_ are exceeded. Although PM_2.5_ concentration levels are significantly higher in more urbanized districts located predominantly in northern India, recent increases have occurred in less urbanized areas located mainly in southern and central India. Multivariable statistical analysis indicated: (1) higher PM_2.5_ concentration in districts with higher percentages of Scheduled Castes (SCs), young children, and households in poor condition residence and without toilets; and (2) higher PM_2.5_ increases in less urbanized districts with higher percentages of SCs, females, children, people with disabilities, and households with no toilets. These findings thus highlight the need to consider the role of air pollution in exacerbating the consequences of social disadvantages in India.

## 1. Introduction

While outdoor air pollution is a major environmental problem across the world, India is estimated to have some of the worst levels globally, especially in terms of fine particulate matter (PM_2.5_) pollution. According to the 2019 Global Burden of Disease, air pollution in India was linked to 1.67 million deaths (17.8% of total deaths), out of which 10.4% were due to ambient PM_2.5_ pollution [1]. This study also calculated that economic losses associated with air pollution-related premature death and morbidity corresponded to 1.36% of India’s Gross Domestic Product (GDP), so that the adverse health implications of air pollution could potentially affect India’s long-term economic targets [1]. A study conducted by the World Health Organization (WHO) covering 100 countries between 2011 to 2016 showed that 14 of the 15 leading cities in terms of PM_2.5_ pollution were located in India [2]. The main contributors to India’s particulate air pollution include industrial and vehicular emissions, construction dust and debris, dependence on thermal power for electricity, waste burning, and use of wood and dung by low-income and rural households for cooking and heating [3]. While shifts to alternative energy sources can potentially address the problem [4], simultaneously occurring upsurges in affluence and intensifications of poverty, as well as lack of effective policies and investment, are likely to ensure the persistence of air pollution [5].

Previous studies of PM_2.5_ pollution have focused on mapping country level distribution patterns and linking them to global burden of disease estimates [6], and have not considered how these patterns relate to demographic characteristics and socioeconomic disadvantages [7]. In the case of India, such studies have examined state and national level patterns of PM_2.5_ pollution [1,8]. Alongside, India’s air pollution has most often been critically analyzed in terms of negative health outcomes or policy pathways that need to be followed to reduce pollution [3,9,10,11,12]. These studies seemingly imply that air pollution is ubiquitous in terms of its social impacts, and thus need to be extended through a consideration of how air pollution’s consequences may unevenly affect specific places and population groups. Distributive environmental justice (EJ), which focuses on analyzing environmental risk burdens faced by socially disadvantaged communities, thus becomes a useful framework for further analysis of socio-spatial patterns of air pollution in India.

This article follows the distributive EJ framework to determine if socially disadvantaged and marginalized communities are disproportionately located in areas burdened by higher particulate air pollution. We measure air pollution on the basis of annual average PM_2.5_ concentration data derived from a combination of satellite observations, a global chemical transport model, and available ground-based measurements [13,14]. Information on population and household characteristics are obtained from the 2011 Census of India. Our unit of analysis is the district since it enables a finer-scale study than the level of the state, while also allowing the use of Census data related to district level characteristics in India. Our key research objectives are to determine if: (a) average PM_2.5_ concentrations in 2010, and (b) increases in average PM_2.5_ concentrations from 2010 to 2016, are distributed inequitably with respect to socially disadvantaged population and household groups, after controlling for relevant contextual factors and clustering in the data. Statistical analyses comprise descriptive comparisons of air quality thresholds established by international and national guidelines, bivariate linear correlations, and multivariable generalized estimating equation (GEE) models.

The aim of this article is to extend research on the distribution and impacts of air pollution in India in three main ways. First, by examining associations between particulate air pollution and social inequalities in India, this study seeks to demonstrate the significance of incorporating environmental injustice into social and environmental policy-making, especially in contexts which combine rising economic growth with persistent and widening social inequities. Second, most international air pollution studies, including WHO and IHME (Institute for Health Metrics and Evaluation) reports, highlight data pertinent to the national scale [15,16]. In the case of India, the focus is on the level of the state or on the most polluted cities [1,2,8]. Our district level analysis thus adds a new spatial dimension to the study of air pollution in India. Third, it is important to consider that while urban growth in India is concentrated in major metropolises, cities just below metropolitan level are also expanding in terms of population and economic growth [2]. A district level study enables a focus on emerging patterns of air pollution in newly urbanizing areas. Overall, this article seeks to address the growing need to examine air pollution in India given its rapid urban growth and major contributions to global economic and environmental outcomes.

## 2. Methods

The two dependent variables for this study—PM_2.5_ pollution in 2010, and change in PM_2.5_ pollution between 2010 and 2016—were estimated using data provided by Van Donkelaar et al. [14]. Explanatory variables were extracted from the 2011 Census of India and included variables that have been widely used in prior distributive EJ studies as well as those relevant to India.

### 2.1. Dependent Variables

Ambient particulate matter pollution was defined for this study as the population-weighted annual average mass concentration of particles with an aerodynamic diameter less than 2.5 micrometers (PM_2.5_) in a cubic meter of air, at a spatial resolution of 0.01° × 0.01° over the globe (approximately 11 × 11 km at the equator). Specifically, we utilized surface PM_2.5_ concentrations estimated by Van Donkelaar et al. [14] using a combination of aerosol optical depth data from multiple satellite products and a global chemical transport model using global emission inventories, subsequently calibrated to ground-based observations of PM_2.5_ using geographically weighted regression (GWR). This methodology and data have been used in several other studies focusing on PM_2.5_ pollution in India [1,3,8,9].

For our analysis, annual mean global GWR-adjusted PM_2.5_ estimates were downloaded from the Atmospheric Composition Analysis Group website [13] as an ArcGIS-compatible NetCDF file. A digital map of districts delineated for the 2011 Census of India was obtained from ML InfoMap/Lead Dog Consulting [17,18] in ArcGIS shapefile format. To calculate our dependent variables for analyzing the distributive EJ implications of PM_2.5_ exposure, the 0.01° × 0.01° resolution grid containing modeled estimates of surface PM_2.5_ concentrations was overlaid on the district boundaries using ArcGIS 10.6.1 software (Environmental Systems Research Institute, Redlands, USA). We then applied the zonal statistics function to determine via areal interpolation the annual average PM_2.5_ concentration for each individual district in 2010 and 2016, based on pixel values from the PM_2.5_ concentration grid.

Our first dependent variable was a district level measure of annual average surface PM_2.5_ concentration in 2010, the year during which our data on population and housing variables were collected for the 2011 Census of India. The second variable represented district level changes in annual average PM_2.5_ concentrations between 2010 and 2016, calculated as a ratio of these two PM_2.5_ concentration values (2016/2010). Our statistical analysis of this second variable includes districts where this ratio exceeded 1.0, following our research focus on increases in PM_2.5_ concentrations in this time-period. We used 2016 values to remain temporally proximate to the 2011 Census of India data while still being able to measure observable change, and also because 2016 was the latest year for which modeled estimates of surface PM_2.5_ concentrations were available in the 0.01° × 0.01° resolution dataset at the time of our analysis [13]. Measurements for our dependent variables are reported in μg/m^3^ and their district level summary statistics are provided in Table 1.

### 2.2. Explanatory Variables

For our distributive EJ analysis of exposure to PM_2.5_ pollution, we used a set of socio-demographic variables from the 2011 Census of India that are available at the district level. Our choice of independent variables to represent socially disadvantaged groups was guided, in part, by previous studies on distributive EJ and social vulnerability to environmental hazards in India [19,20,21,22]. Independent variables were derived from two sources: the 2011 Census of India Primary Census Enumeration data and the Houselisting and Housing Census data [23,24]. District level summary statistics for independent variables are provided in Table 1.

Variables measuring socioeconomic disadvantage included illiteracy rate and caste and tribal status, and these have also been utilized in previous distributive EJ studies [19,21,22]. Literacy rate, the proportion of population seven years of age or above that can undertake basic reading and writing, has often been used as a proxy for socioeconomic status, and was utilized to ascertain the level of development at the district level. Literacy can shape the ability of a population to either accept the presence of pollution as a corollary to their own economic development, or challenge the presence of pollution due to access to effective political power. To reflect disadvantage, we used illiteracy rates as the independent variable in our study. The proportion of population belonging to Scheduled Caste (SC) and Scheduled Tribe (ST) groups were used to represent the two main socially marginalized groups in India who are formally listed in the Indian Constitution. The marginalization of SC groups reflects discrimination against lower castes in India due to their traditional means of livelihood and their exclusion from sources of ritual power. The designation ST refers to social groups that have maintained a distinctive culture, often through forest or natural resources-dependent livelihoods. While SCs belong to Hindu, Buddhist, and Sikh religious communities, STs can belong to any religious group. Previous national level research indicates that SC percentages are significantly higher in Indian districts that generate industrial hazardous waste, compared to those that do not produce such waste [21].

The percentages of the district population who are female, aged six years or less, and with a disability were included to examine the exposure of demographically and biologically vulnerable groups to PM_2.5_ pollution. Although gender has been relatively less utilized in the distributive EJ research literature, the prevalence of a masculine sex ratio in northern India makes gender a useful component of social exposure to pollution here. Children’s exposure to hazards has also been relatively understudied in India, but has been an important area of focus in U.S. EJ studies [25,26,27]. Children are considered to be more vulnerable to air pollution than adults due to their higher breathing rate to body size ratio, and the still developing nature of their lungs [28]. Distributive EJ studies in the U.S. have found neighborhoods with higher proportions of children to have significantly higher levels of exposure to industrial hazards [25,26,29,30]. The proportion of population with disabilities was selected as an additional explanatory variable. Although the relationship between disability and air pollution exposure has not yet been investigated in India, recent studies in the U.S. have found people with disabilities to reside in neighborhoods facing significantly higher exposure to various pollution sources than people without disabilities [31,32]. Our study seeks to examine whether these patterns also emerge in the case of India.

In addition to the aforementioned variables from the Primary Census Enumeration, we utilized four indicators of socioeconomic disadvantage from the 2011 Houselisting and Housing Census. These were the proportion of households without availability of any assets such as a television, computer/laptop, telephone/mobile phone, or a scooter/car; residing in a house whose condition was not ‘good’ (either ‘dilapidated’ or ‘livable’); whose drinking water source is not located within their premises (‘near premises’ or ‘away from house’); and with no toilet or latrine facility within their premises. These variables have been used to represent social and economic disadvantage in previous scholarship on environmental hazards in India [20] and are likely to be linked to increased intensity of exposure to air pollution (due to poor housing quality and amenities) and reduced ability to cope with air pollution (due to lower economic status).

Finally, population density and the proportion of urban population in the district were used as control variables for our analysis, following previous EJ studies that have used both these variables [21,22]. The proportion of urban population in India is calculated based on the population residing in census towns or statutory towns. Census towns have a population of at least 5000 people, a density of population of at least 400 people per square kilometer, and at least 75% of main male workers engaged in non-agricultural occupations. Statutory towns are administered by a municipality, corporation, cantonment board, or notified area committee. Previous studies have shown that pollution-generating activities in India often locate in low-density or sparsely populated areas adjacent to densely populated urban centers, so that they can take advantage of higher availability of vacant land that remains accessible and proximate to large urban areas [21,22]. Overall, our set of independent variables represents social and economic characteristics that are most relevant for understanding the injustices associated with exposure to particulate air pollution.

### 2.3. Statistical Analyses

Our study began by identifying districts where the annual average PM_2.5_ concentration in 2010 exceeded air quality standards or thresholds recommended by the World Health Organization (WHO), U.S. Environmental Protection Agency (USEPA), European Union (EU), and India’s National Ambient Air Quality Standards (NAAQS). We then estimated the overall proportions of population and households, as well as the proportions of socially disadvantaged population and households (as a percentage of their total numbers in India), residing in districts where ambient PM_2.5_ pollution levels exceeded these thresholds.

Bivariate correlation analysis was conducted for an initial exploration of statistical relationships between each explanatory variable and the two dependent variables: annual average PM_2.5_ concentrations (2010) based on all 640 districts in India; and ratio of PM_2.5_ concentrations (2016/2010) based on 601 districts that experienced an increase in PM_2.5_ concentrations (i.e., ratio of 2016/2010 PM_2.5_ concentrations > 1.0).

We then used generalized estimating equations (GEEs), a multivariable modeling technique appropriate for analyzing clustered data, to examine the EJ implications of both PM_2.5_ (2010) concentrations and increase in PM_2.5_ concentration ratio (2016/2010). GEEs relax several assumptions of traditional regression models and impose no strict distributional assumptions for the included variables, in addition to accounting for clustering of variables across units of analysis. In the case of India, districts are clustered within 35 states or union territories (UTs). Our clustering definition was thus based on the state or UT within which each district was located, which led to the assignment of 1 to 71 districts within each state/UT cluster.

A separate GEE was estimated for each of our two dependent variables: annual average PM_2.5_ concentration in 2010 (640 districts), and ratio of PM_2.5_ concentrations: 2016/2010 (601 districts where ratio > 1.0). Although the second GEE utilizes both 2010 and 2016 PM_2.5_ data, the explanatory variables are based on data from 2010, or the beginning of the period over which PM_2.5_ increase is calculated. Potential endogeneity among explanatory variables in a multivariable model can be reduced by measuring these variables at the beginning of the time-period over which change is calculated [33] and this approach has been recommended in prior econometric studies on modeling temporal change [34,35,36].

For each GEE, three different correlation structure specifications were considered [37]: ‘independent’, which assumes the nonexistence of dependency so that all off-diagonal elements of the working correlation matrix are zero; ‘exchangeable’, which assumes constant intra-cluster dependency so that all the off-diagonal elements of the correlation matrix are equal; and ‘unstructured’, which assumes a completely general correlation matrix that is estimated without constraints. All GEEs were modeled with the three matrices, using the QIC (quasi-likelihood under the independence model criterion) to determine the most appropriate specification. Using this model fit criterion, we chose the ‘independent’ correlation matrix for the GEE using the average PM_2.5_ concentration (2010) as the dependent variable, and ‘unstructured’ for the GEE using the PM_2.5_ concentration ratio (2016/2010) as the dependent variable.

To select the best-fitting models, we tested the normal, gamma, and inverse Gaussian distributions with logarithmic and identity link functions [37]. An identity link function assumes the dependent variable is directly predicted, while a logarithmic link function estimates the natural logarithm of the dependent variable. We selected the gamma distribution with logarithmic link function for the GEE using the average PM_2.5_ concentration (2010) as the dependent variable, and the normal distribution with an identity link function for the GEE where the PM_2.5_ concentration ratio (2016/2010) was used as the dependent variable. Both these model specifications yielded the lowest value of the QIC.

All independent variables were standardized before inclusion in the GEEs. We also checked for potential multicollinearity among these variables using variance inflation factor, tolerance, and condition index criteria, and confirmed that the GEEs are not affected by multicollinearity. Two-tailed p-values from the Wald chi-square test were used to evaluate the statistical significance of each individual variable coefficient.

## 3. Results

**Figure 1:**
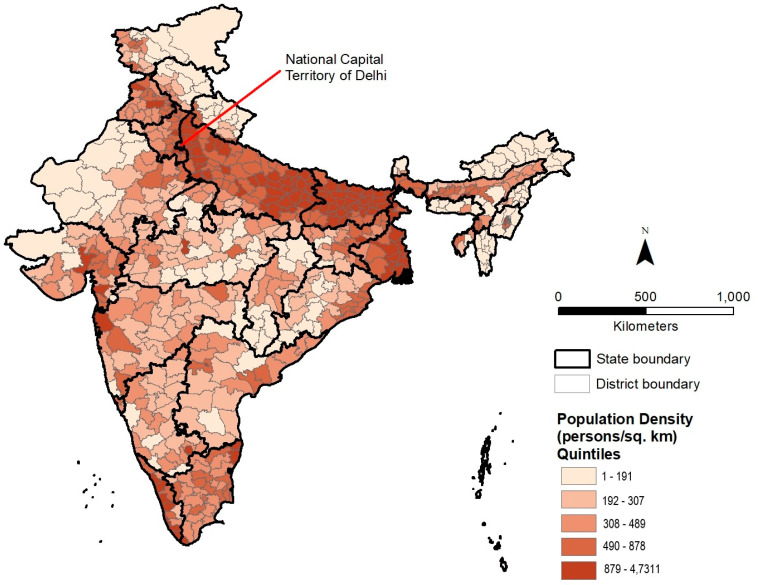
Distribution of 2011 population density by district in India.

To provide a geographic context for comparing and understanding the spatial patterns of our air pollution-related dependent variables, the district level distribution of population density is first presented in Figure 1. On this map, districts in India are classified into five quintiles based on the number of people per square km. Districts with the highest population density values are located primarily in a multi-state region across northwest to eastern India known as the Indo-Gangetic Plain (IGP). One of the most densely populated regions of the world, the IGP is also India’s major agricultural belt due to the Ganges and Yamuna rivers. Additionally, 12 of the 14 Indian cities that are among the world’s 20 most polluted in terms of PM_2.5_ levels are located in the IGP [2].

**Figure 2:**
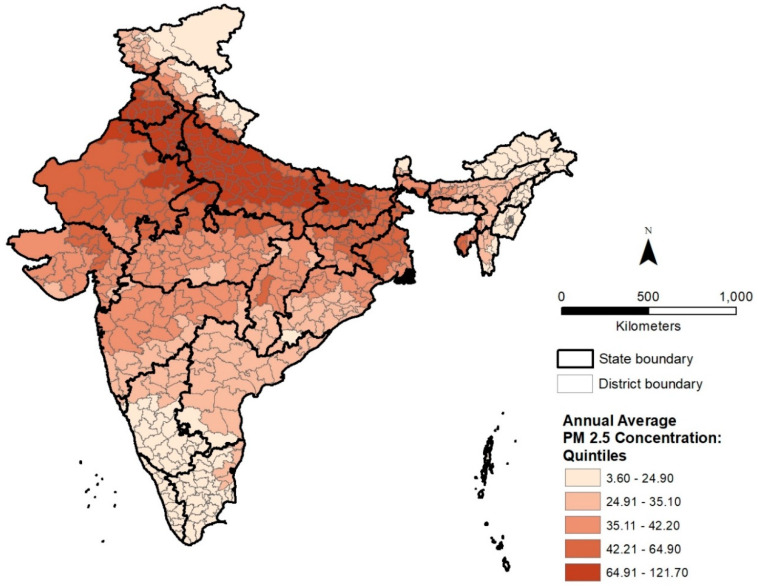
Distribution of 2010 surface annual average PM_2.5_ concentrations (μg/m^3^) by district in India.

The geographic distribution of ambient surface PM_2.5_ pollution in 2010 is depicted in Figure 2, where districts are classified into five quintiles based on annual average PM_2.5_ concentrations. The district level distribution of PM_2.5_ pollution suggests a strong spatial correspondence with the distribution of population density shown in Figure 1, with districts in the highest quintile (top 20%) concentrated mainly in the IGP. Six of the top ten districts are located in the National Capital Territory (NCT) of Delhi, with the remaining in its surrounding states of Uttar Pradesh (three districts) and Haryana (one district). If Delhi’s districts are excluded, eight of the top ten districts are located in the state of Uttar Pradesh and two in Haryana, again revealing the high levels of air pollution in the IGP. In contrast, districts in the lowest quintile (bottom 20%) of PM_2.5_ concentration can be found primarily in southern India, northeast India, and northern India. Higher population density, prevalence of coal mines, brick kilns, and power plants, as well as meteorological factors have been used to explain the disparities in air pollution levels between the land locked IGP and southern India [3]. Lower particulate pollution levels in northeast and northern India can be linked to lower levels of industrialization in these regions.

**Figure 3:**
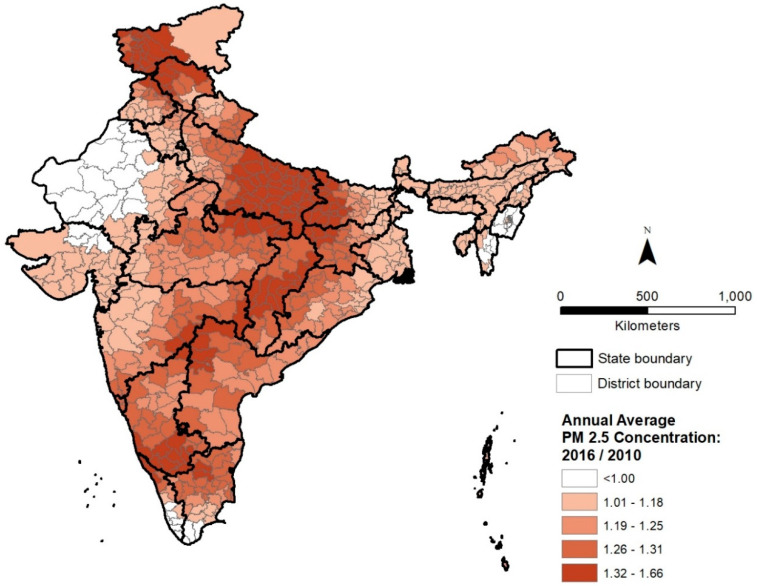
Distribution of 2016/2010 ratio of annual average PM_2.5_ concentrations (μg/m^3^) by district in India.

The district level distribution of average PM_2.5_ concentration ratio (2016/2010) is shown in Figure 3. The geography of recent changes in PM_2.5_ pollution differs substantially from the overall pattern of PM_2.5_ pollution in 2010 and suggests a more dispersed spatial pattern compared to Figure 2. The district level distribution of PM_2.5_ increase also does not indicate a strong spatial correspondence with the distribution of population density depicted in Figure 1. Although several districts in the highest quintile of PM_2.5_ concentration ratio (2016/2010) can be observed in the IGP, other districts in this quintile are located in southern, central, and northern India. Figure 3 suggests substantially high increases in PM_2.5_ pollution in several southern districts, especially along a belt containing large cities such as Bengaluru and Hyderabad, both major hubs for information technology industries. The increase in air pollution at India’s northern border could also be associated with increasing urbanization. In central India, increases coincide spatially with a belt of coal mining and associated industrial activities. Districts where PM_2.5_ concentrations have declined since 2010 are located mainly in the states of Rajasthan in the northwest, Manipur and Mizoram in the northeast, and Kerala in the south because of lower levels of industrialization.

We began our statistical analysis by estimating the population and household characteristics of districts where the average PM_2.5_ pollution levels in 2010 exceeded international and national air quality standards. These results are summarized in Table 2.

Only about 0.04% of India’s population and 0.03% of households reside in districts where PM_2.5_ concentrations recommended by the WHO (10 μg/m^3^) are not exceeded, and 0.08% of population and households where USEPA standards (12 μg/m^3^) are not exceeded. These percentages increase to around 13% of population and 15% of households when the EU standards (25 μg/m^3^) are used. Districts where PM_2.5_ pollution exceeds all these international standards were found to contain at least 85% of the population and households associated with the socially disadvantaged categories we examined. However, about 56% of the population and 51% of households can still be found in districts where average PM_2.5_ concentration exceed the higher national threshold (40 μg/m^3^) used in India. The percentages of illiterates, SCs, and children in these districts exceed the overall population percentage (55.6%), while the percentages of households in poor condition houses and households without toilets exceed the overall percentage of households (51.3%). However, the percentages of STs, females, and people with disabilities, as well as households without assets and households with drinking water source outside their premises are lower than the overall population and household percentages in districts where India’s PM_2.5_ standards are exceeded.

To analyze the statistical effects of our explanatory variables on 2010 PM_2.5_ concentrations and 2016/2010 PM_2.5_ concentration ratios, we first utilized bivariate linear correlations. Pearson’s product-moment correlation coefficients associated with each pair of variables are presented in Table 3. PM_2.5_ concentration (2010) is significantly and positively correlated with population density and percentages of urban population, illiterates, SCs and children, as well as households in poor condition residence and without toilets. Variables showing a significant negative correlation with PM_2.5_ pollution include the percentages of female and ST population, as well as households with no assets and outside drinking water source. PM_2.5_ concentration ratio (2016/2010) in districts with increase is significantly and positively associated with the percentages of SCs, children, and households without toilets, and negatively associated with the percentages of STs and households with no assets; these results align with PM_2.5_ pollution correlations. Population density, female percentage, and percentage of households in poor condition residence and with outside drinking water source indicate a non-significant correlation with PM_2.5_ increase, despite their significant negative associations with PM_2.5_ pollution levels. Although the percentage of people with disabilities showed a non-significant association with PM_2.5_ pollution, it is significantly and positively correlated with PM_2.5_ increase.

The results of our multivariable GEE models are summarized in Table 4 and Table 5. Table 4 indicates that, after controlling for the effects of clustering and other relevant factors, average PM_2.5_ concentrations (2010) are significantly greater in densely populated districts with higher percentages of urban, SC population, and children, as well as higher proportions of households residing in poor condition residences and those without toilets. The percentages of female population and households with outside drinking water source are the only variables to indicate a significantly negative association with PM_2.5_ concentration.

With regards to PM_2.5_ concentration ratio (2016/2010), Table 5 indicates significantly greater PM_2.5_ increases in districts with higher percentages of SCs, females, children, and people with disabilities, as well as households with no toilets. Greater PM_2.5_ pollution increases are also significantly associated with lower percentages of urban and illiterate populations, households without assets, and those in poor condition residences.

## 4. Discussion

Our preliminary statistical analysis drew attention to the range of international and national standards on PM_2.5_ pollution and districts in India where these air quality standards were exceeded (Table 2). Districts with higher outdoor PM_2.5_ concentrations in 2010 than recommended international guidelines were found to contain more than 85% of India’s population and households. At least 85% of people and households associated with each of our socially disadvantaged groups are also located in these districts. Districts where PM_2.5_ pollution exceed India’s national air quality standards contain about 56% and 51% of those in our socially disadvantaged population and household categories, respectively, with the sole exception of the ST population. As found in previous research, the presence of ST populations in less industrialized areas of northeastern and northern India implies that they are also located in areas with lower particulate air pollution [21].

Our primary research goal was to determine if PM_2.5_ pollution and its recent increases were significantly greater in districts with higher proportions of socially disadvantaged groups, after controlling for spatial clustering, population density, and other contextual factors. Our multivariable GEE analysis revealed higher PM_2.5_ concentration in urbanized districts with higher percentages of SCs, young children, households in poor condition residence, and households without toilets. We also found significantly higher PM_2.5_ concentration increases in less urbanized districts with higher percentages of SCs, female, children, and people with disabilities, as well as households with no toilets.

Overall, our results provide strong evidence of significantly higher air pollution risk burdens for specific disadvantaged and vulnerable population groups. Both bivariate and multivariable statistical analyses indicated that SCs and young children (0–6 years) are disproportionately located in districts with higher exposure to PM_2.5_ pollution, as well as those with the highest increases in PM_2.5_ pollution. These findings emphasize the urgent need for addressing and reducing PM_2.5_ emissions, since SCs as a socially marginalized group often lack access to protective resources or are unable to afford risk mitigation, and children are physically more susceptible to adverse health effects of such emissions. The female percentage was significantly lower in districts with higher PM_2.5_ concentration, but greater in districts with higher PM_2.5_ increases. The negative association with PM_2.5_ pollution is connected to higher male population in larger urban areas and districts of IGP, while the positive association can be explained by PM_2.5_ increases in southern states and medium sized cities with relatively higher female proportions. These findings could also indicate a male pattern of urban migration in India, so that the most polluted areas also offer greater employment opportunities for migrant men. However, as a secondary-tier of urban settlements begin to show the effects of economic and industrial development, the proportion of women facing exposure to PM_2.5_ pollution has also increased.

In contrast to the SC population, STs are significantly underrepresented in the most polluted districts based on our bivariate analysis, and also show a non-significant association in our multivariable models. This finding can be explained by their lower proportions in districts of the IGP and other regions characterized by higher PM_2.5_ pollution and recent increases. It could also reflect lack of economic or industrial development in districts in northeastern and extreme northern states with higher ST proportions that potentially lead to lower PM_2.5_ levels in these areas. Our multivariable analysis indicated that the percentage of people with disabilities is significantly higher in districts with greatest PM_2.5_ increases, but not significantly associated with PM_2.5_ exposure. This finding could reflect a pattern similar to women whereby people with disabilities are not found in highly urban areas since they may not be able to seek industrial employment, but as air pollution begins to increase in medium sized cities, they too face increasing exposure. The illiterate population was found to be overrepresented in districts with the highest concentrations and increases in PM_2.5_ pollution in our bivariate analysis. However, after controlling for additional socioeconomic variables in our multivariable models, illiteracy rate indicated a non-significant relationship with PM_2.5_ exposure and a significantly negative relationship with PM_2.5_ increase. The latter could be potentially associated with significant increases of PM_2.5_ concentrations in several districts of southern India with higher literacy rates.

Our findings also reveal housing quality-related injustices in the distribution and increase of PM_2.5_ pollution. Both bivariate and multivariable analyses indicate significantly greater PM_2.5_ exposure and increases in districts with a higher percentage of households without toilets. This could be partially related to their relatively higher proportions in states of the IGP and central India. Similar positive associations were observed for the percentage of households residing in poor condition residence in bivariate analyses and multivariable analysis of PM_2.5_ exposure. However, the negative coefficient for this variable in the multivariable model for PM_2.5_ increase could potentially be linked to relatively lower proportions of households in poor condition houses in districts of south India which experienced recent PM_2.5_ increases. The percentage of households without assets was not significantly related to PM_2.5_ levels and negatively related to PM_2.5_ increases in our multivariable analysis. This is not entirely surprising because of the lower proportions of this group in densely populated and urbanized districts of the IGP and southern India, which experienced substantially higher PM_2.5_ exposure and increases. Similar location patterns have influenced the statistical results for the proportion of households with drinking water outside their premises, which was found to be negatively and non-significantly related to PM_2.5_ concentration and increase. Overall, these findings show that while asset ownership and access to drinking water have increased in urban India, quality of house construction and access to toilets still remains a matter of concern, particularly in areas of higher air pollution levels and recent increases.

## 5. Conclusions

Our distributive EJ analysis reveals geographically and socially disparate patterns of exposure to ambient PM_2.5_ pollution, as well as recent increases in PM_2.5_ pollution, at the district level in India. Districts with higher percentages of SCs, children, and households without toilets are burdened by significantly higher levels of both surface PM_2.5_ and recent increases in PM_2.5_, even after controlling for clustering and contextual factors. In addition to these three indicators of social disadvantage or vulnerability, the proportion of households in poor condition residences are significantly greater in districts with higher PM_2.5_ pollution, while the proportion of people with disabilities are significantly greater in districts with higher PM_2.5_ increases. Although PM_2.5_ exposure is significantly higher in more urbanized districts located mainly in the IGP region, PM_2.5_ exposure indicated a recent increase in relatively less urbanized areas located predominantly in southern and central India. These differences in spatial patterns potentially suggest increasing air pollution in medium or small Indian cities that could be driven by migration and regional population shifts, as well as by industries moving towards less urbanized areas to take advantage of lower levels of pollution. This pattern of increase also suggests that besides SCs and children, women and people with disabilities are also facing increasing exposure to air pollution thus expanding the characteristics of vulnerable populations facing environmental injustices in India. While our study provides an important starting point, additional analyses and data sources are necessary to examine how factors such as population growth, rural-urban migration trends, and changing patterns of economic and industrial development have contributed to increases in PM_2.5_ pollution and their unequal social impacts.

As air pollution becomes a tangible concern across urban India, it is expected that citizen pressure will move government and business interests towards privileging policies and practices that protect environmental quality [38]. These could include stricter regulation of pollution by the Central and State Pollution Control Boards and a shift to less polluting transport options, as well as greater investment in ‘green’ technologies, renewable energy, and effluent treatment by industrial units [4]. However, there may also be a tendency to form ‘pollution havens,’ further exacerbating the exposure to pollution faced by socially disadvantaged groups and enabling more advantaged segments to relocate to less polluted enclaves. Alongside, an unmitigated emphasis on economic growth is leading India to relax environmental laws in an attempt to draw foreign investment. There is thus a broader global context within which the economic incentive to pollute is high enough to cause corporations and governments to disregard their social costs. Within this framework, the work of local activists and national environmental organizations becomes that much more challenging, especially when upper and middle classes have the option to move away from sites of pollution. Much remains to be done then to align EJ objectives with profit-making pressures.

This study has drawn attention to the relationship between increasing particulate air pollution and increasing environmental injustices in India, and through this to emphasize the need to delve further into the social consequences of worsening environmental quality. Future studies need to consider how exposure to other forms of pollution, including water and soil pollution, reveal similar forms of injustices. Moreover, the ways in which multiple forms of pollution intersect with each other and with social disadvantages needs to be studied in more detail. Particulate pollution is one element of a larger set of negative pollution outcomes, and while its severity signals the urgent need to control polluting activities, it should also be viewed as the leading edge of a more comprehensive objective to tackle India’s environmental injustices by improving environmental quality.

## Figures and Tables

**Table 1:** Summary statistics for variables analyzed (unit of analysis: district).

Variables	*N*	Min.	Max.	Mean	SD
**Dependent:**					
Surface annual average PM_2.5_ 2010 (μg/m^3^)	640	3.60	121.70	45.03	24.38
Increase: PM_2.5_ 2016 (μg/m^3^) / PM_2.5_ 2010 (μg/m^3^)	601	1.00	1.65	1.22	0.13
**Independent:**					
Population density (persons per square km)	640	1	47,311	1009	3697
% Urban population	640	0.00	100.00	26.40	21.12
% Illiterate population (more than 6 years)	640	12.25	89.37	43.92	13.58
% Scheduled Caste population	640	0.00	50.17	14.86	9.13
% Scheduled Tribe population	640	0.00	98.58	17.63	26.87
% Female population	601	40.84	54.22	48.53	1.52
% Children (6 years or less)	640	5.47	23.01	13.89	2.95
% People with disabilities	640	0.76	4.51	2.15	0.57
% Households (HHs) with no assets *	640	1.00	64.90	20.38	12.93
% HHs in poor condition residence	640	13.30	87.20	50.69	14.30
% HHs with drinking water source outside premises	640	6.10	97.60	57.65	22.95
% HHs with no toilet	640	1.10	94.40	53.63	26.30

* HH assets include television, computer/laptop, telephone/mobile phone, and/or car/scooter.

**Table 2:** Percentages of population and household subgroups in India where 2010 annual average PM_2.5_ concentrations exceeded standards established by the World Health Organization (WHO), U.S. Environmental Protection Agency (USEPA), European Union (EU), and India.

Variables	WHO: >10 μg/m^3^	USEPA: >12 μg/m^3^	EU: >25 μg/m^3^	India: >40 μg/m^3^
Districts (640 total)	632	627	510	291
Total population	99.96%	99.92%	87.04%	55.59%
Urban population	99.96%	99.92%	82.74%	51.60%
Illiterate population	99.96%	99.92%	90.28%	60.08%
Scheduled Caste population	99.99%	99.99%	88.63%	61.98%
Scheduled Tribe population	99.62%	99.39%	89.98%	32.95%
Female population	99.96%	99.92%	86.62%	54.82%
Children (6 years or less)	99.96%	99.92%	89.68%	60.47%
People with disabilities	99.95%	99.91%	88.39%	54.48%
Total households (HHs)	99.97%	99.92%	84.99%	51.28%
% HHs with no assets	99.97%	99.92%	91.58%	49.92%
% HHs in poor condition residence	99.97%	99.93%	88.80%	57.26%
% HHs with drinking water sourceoutside premises	99.96%	99.92%	85.08%	48.01%
% HHs with no toilet	99.99%	99.96%	88.89%	55.67%

**Table 3:** Bivariate linear correlations of average PM_2.5_ concentration (2010) and PM_2.5_ concentration ratio (2016/2010) with district level characteristics.

Variables	PM_2.5_: 2010		PM_2.5_: 2016/2010	
	Pearson’s *r*	*p*-Value	Pearson’s *r*	*p*-Value
Population density	0.251	<0.01	−0.078	0.057
% Urban population	0.103	<0.01	−0.169	<0.01
% Illiterate population	0.190	<0.01	0.155	<0.01
% Scheduled Caste population	0.407	<0.01	0.323	<0.01
% Scheduled Tribe population	−0.399	<0.01	−0.295	<0.01
% Female population	−0.473	<0.01	0.071	0.083
% Children (6 years or less)	0.255	<0.01	0.095	<0.05
% People with disabilities	−0.072	0.068	0.179	<0.01
% HHs with no assets	−0.257	<0.01	−0.214	<0.01
% HHs in poor condition residence	0.227	<0.01	0.024	0.556
% HHs with drinking water source outside premises	−0.336	<0.01	0.029	0.481
% HHs with no toilet	0.081	<0.05	0.347	<0.01
*N* (districts)	640		601	

**Table 4:** Generalized Estimating Equation (GEE) for predicting average PM_2.5_ concentration in 2010 (*N* = 640).

Variables	Standardized Beta	Standard Error	Lower 95% CI	Upper 95% CI	Wald chi-square	*p*-Value
Population density	0.052	0.017	0.019	0.085	9.644	<0.01
% Urban population	0.066	0.039	−0.010	0.142	2.908	<0.05
% Illiterate population	−0.063	0.046	−0.154	0.028	1.865	0.172
% Scheduled Caste population	0.169	0.035	0.099	0.238	22.648	<0.01
% Scheduled Tribe population	−0.059	0.038	−0.132	0.015	2.468	0.116
% Female population	−0.129	0.040	−0.208	−0.050	10.197	<0.01
% Children (6 years or less)	0.232	0.053	0.128	0.337	18.974	<0.01
% People with disabilities	−0.025	0.030	−0.085	0.034	0.696	0.404
% HHs with no assets	−0.047	0.043	−0.132	0.037	1.198	0.274
% HHs in poor condition residence	0.091	0.038	0.016	0.165	5.735	<0.05
% HHs with drinking water source outside premises	−0.143	0.044	−0.228	−0.058	10.812	<0.01
% HHs with no toilet	0.112	0.043	0.028	0.196	6.889	<0.01
Intercept	3.722	0.045	3.633	3.810	6833.022	<0.01
Scale	0.103					
Model fit (QIC)	213.831					

Note: GEE is based on a gamma distribution with log link function and independent correlation matrix.

**Table 5:** GEE for predicting 2016/2010 PM_2.5_ concentration ratio (*N* = 601).

Variables	Std. Beta	Std. Error	Lower 95% CI	Upper 95% CI	Wald chi-square	*p*-Value
Population density	0.001	0.005	−0.008	0.011	0.087	0.768
% Urban population	−0.023	0.007	−0.037	−0.010	11.121	<0.01
% Illiterate population	−0.040	0.014	−0.067	−0.014	8.800	<0.01
% Scheduled Caste population	0.023	0.010	0.003	0.043	5.105	<0.05
% Scheduled Tribe population	0.014	0.009	−0.003	0.031	2.780	0.095
% Female population	0.016	0.008	0.000	0.033	3.865	<0.05
% Children (6 years or less)	0.063	0.011	0.041	0.086	31.137	<0.01
% People with disabilities	0.028	0.007	0.014	0.042	15.509	<0.01
% HHs with no assets	−0.071	0.013	−0.096	−0.046	29.919	<0.01
% HHs in poor condition residence	−0.035	0.012	−0.058	−0.011	8.248	<0.01
% HHs with drinking water source outside premises	−0.010	0.012	−0.034	0.014	0.675	0.411
% HHs with no toilet	0.075	0.012	0.051	0.098	38.350	<0.01
Intercept	1.228	0.010	1.207	1.248	13,920.182	<0.01
Scale	0.011					
Model fit (QIC)	88.625					

Note: GEE is based on a normal distribution with identity link function and unstructured correlation matrix.
